# A Structural Equation Model of Perceived Autonomy Support and Growth Mindset in Undergraduate Students: The Mediating Role of Sense of Coherence

**DOI:** 10.3389/fpsyg.2020.02055

**Published:** 2020-09-03

**Authors:** Chunhua Ma, Yongfeng Ma, Xiaoyu Lan

**Affiliations:** ^1^ College of Educational Science and Technology, Northwest Minzu University, Lanzhou, China; ^2^ Department of Developmental Psychology and Socialization, University of Padova, Padova, Italy

**Keywords:** parental autonomy support, teacher autonomy support, sense of coherence, growth mindset, undergraduate students

## Abstract

Although prior research has extensively documented the correlates of growth mindset, little is known about its antecedents in undergraduate students. Guided by the self-determination theory, the current study investigated the association of perceived autonomy support (i.e., parental autonomy support and teacher autonomy support) with growth mindset and assessed whether sense of coherence mediated this association. A total of 1,030 Chinese undergraduate students (62.4% females; *M*_age_ = 20.44, *SD* = 1.52) aged from 18 to 25 years were involved in this study; they were asked to fill out a set of self-reported questionnaires. Results of the structural equation modeling showed that sense of coherence fully mediated the association between parental autonomy support and growth mindset and between teacher autonomy support and growth mindset. More precisely, parental autonomy support and teacher autonomy support were each positively associated with sense of coherence, which in turn was positively related to growth mindset. The current findings further confirm the beneficial effect of autonomy support on individuals’ adaptive skills in a collective cultural context, suggesting that autonomy-supportive parents and teachers can contribute to undergraduate students’ growth mindset through the role of sense of coherence.

## Introduction

Growth mindset concerns the belief that an individual’s basic attributes are malleable and can be cultivated through endeavors (Dweck et al., 1995; Dweck, 2015). Recently, a burgeoning body of research has demonstrated that high levels of growth mindset are positively related to optimal functions across the psychological, social, and academic domains, such as psychological well-being (Zeng et al., 2016), less mental health difficulties (Schleider et al., 2015; Schleider and Weisz, 2018; Wang et al., 2019), prosocial tendency (Han et al., 2018), self-regulation (Burnette et al., 2013; Lan et al., 2019a), learning motivation (Burnette et al., 2018), and academic achievement (Yeager et al., 2016; Wang et al., 2020). Given the salience role of growth mindset in life courses, the knowledge about the contextual and individual antecedents of growth mindset is still limited in existing research. Several salient features of the Chinese society make it an especially appropriate context in which to investigate the antecedents of growth mindset.

As described in a well-known Chinese proverb, “If you work hard enough, you can grind an iron rod into a needle” (只要功夫深, 铁杵磨成针; Curdt-Christiansen, 2008). Indeed, embedded in Confucian ideology, the Chinese culture underscores the belief that intrinsic motivation, continuous endeavors, and willpower are essential to personal success (Lan et al., 2019b; Ma et al., 2020), which are independent of individuals’ intelligence and social status (Curdt-Christiansen, 2008). Moreover, the importance of persistence and determination is especially highlighted during critical life transitions, such as undergraduate students. With the rapid economic development and fierce competition in modern Chinese society, Chinese undergraduate students have to contend with many difficult challenges, such as job seeking or the pursuit of higher education (Arnett, 2000; Smith and Yang, 2017; Lan et al., 2019a). Therefore, a better understanding of the antecedents of growth mindset among Chinese undergraduate students would be crucial as it may impact their academic performance in college, future career ambitions, and psychological well-being in later life.

To concisely summarize, the present research aimed to examine the association of perceived autonomy support (parental autonomy support and teacher autonomy support) with growth mindset and to explore whether sense of coherence mediated this association among Chinese undergraduate students. In the following sections, we conducted an up-to-date literature review to figure out the possible associations among the study variables, starting from the presentation of perceived autonomy support.

### Perceived Autonomy Support and Growth Mindset

According to the self-determination theory (SDT; Ryan and Deci, 2000), autonomy, as one of the basic psychological needs (i.e., relatedness, autonomy, and competence), refers to the experience of choice and psychological freedom concerning one’s activities. In line with this tenet, autonomy support involves an interpersonal style in which parents or teachers take the perspective of their children or students into account, present rationales for demands, acknowledge their feelings, and provide opportunities for choice and self-determination (Ryan and Deci, 2000; Jungert and Koestner, 2015). Although testing the cross-cultural generalizability of SDT has been proposed for a few decades, relatively fewer empirical investigations have been devoted to ascertaining this issue (Ryan and Deci, 2000; Vansteenkiste et al., 2008). According to recent cross-cultural studies conducted among US and Indian adults, Miller et al. (2011) have shown that the need for choice is as important in individualistic as in collectivistic culture, which supports the universality of choice in agency across distinct cultures. In contrast, this tenet is often challenged by some theorists, arguing that autonomy support is critical only for those who live in cultures where autonomy is explicitly valued (e.g., Markus and Kitayama, 1991, 2003; Vansteenkiste et al., 2005). Despite an increasing awareness of this issue (Nalipay et al., 2020), considering significant sociocultural changes in the past decades in China, some research attention still should be paid to clarifying the beneficial effect of perceived autonomy support on undergraduate students’ adaptive skills.

Furthermore, SDT addresses that individuals are inherently active, and intrinsic motivations emerge automatically in his or her interaction with the environment; if available, social support resources can foster their autonomy, thereby activating their organismic processes of growth and integration (Ryan and Deci, 2000; Jungert et al., 2015). In this regard, encouraging individuals to explore independently and validate their perspectives may engender a sense of control and mastery, which may channel them into beliefs that basic attributes are malleable and can be cultivated through efforts (Kim et al., 2017).

From an empirical perspective, Kim et al. (2017) have found that parental autonomy support is positively related to growth mindset in Chinese youth. More recently, a similar positive association between parental autonomy support and growth mindset has been found in a sample of Chinese undergraduate students (Lan et al., 2019a). Despite such evidence, there are relatively limited empirical studies concerning teachers’ beliefs about self-determination on undergraduate students’ adaptive skills. Given that undergraduate students spend most of their time in college, teachers may become the more prominent autonomy-supporting figure, which deserves further research attention.

Moreover, much of the research has examined the association between a separate form of perceived autonomy support (e.g., parental autonomy support or teacher autonomy support) and psychosocial functions (see meta-analyses by Núñez and León, 2015; Vasquez et al., 2016). Indeed, among all the significant others in individuals’ daily life, autonomy-supportive parents and teachers^1^ may provide this opportunity, given their critical roles in the development of self-determination for individuals (Chirkov and Ryan, 2001; Jungert and Koestner, 2015). For example, by simultaneously investigating autonomy support from both parents and teachers, Jungert and Koestner (2015) have found that teacher autonomy support is positively related to motivation, self-efficacy, and achievement over time, while parental autonomy support is not directly related to these outcomes for students during their final year of high school. However, little is known about whether autonomy support from different aspects has similar or distinct effects on growth mindset in one single investigation. In addition, little attention has been paid to ascertaining the underlying mechanism between perceived autonomy support and growth mindset. In this study, we assumed that sense of coherence might fill this knowledge gap.

### The Mediating Role of Sense of Coherence

Sense of coherence refers to a global orientation that individuals view the world and life events as comprehensible, believe that they have sufficient resources to cope with life stressors, and that the efforts they exerted are meaningful (Antonovsky, 1987). Sense of coherence is regarded as a stable trait that is rapidly developed in young adulthood and stabilizes around the age of 30 (Schnyder et al., 2000; Super et al., 2016). According to prior investigation, individuals with a high sense of coherence are cognitively and emotionally capable of handling life difficulties, thereby exhibiting an adaptive psychological functioning (e.g., less mental health difficulties and greater psychological well-being; see Eriksson and Lindström, 2007, for a review). In the current study, we proposed that sense of coherence mediated the association between perceived autonomy support and growth mindset based on the salutogenic theory (Antonovsky, 1987; Mittelmark et al., 2017). As demonstrated by this theory, the presence of supportive social contexts enhances individuals to view stressful life events as comprehensible, manageable, and meaningful. In this regard, individuals are better able to understand the stressors and to accept the challenges (Super et al., 2016; Swan et al., 2018). It is conceivable that a high sense of coherence may enable an individual to pay continuous efforts to fight against life difficulties and challenges, thereby yielding a malleable belief.

From an empirical perspective, using a clinical sample, Langeland and Wahl (2009) have demonstrated that perceived social support can predict adults’ positive development in sense of coherence. Recently, this association has been confirmed by other empirical investigations (e.g., Pasek et al., 2017). Taken together, these empirical studies indicate that the quality of social support, such as perceived autonomy support, may positively enhance undergraduate students’ sense of coherence.

### The Present Study

The purpose of the current study was to investigate the association between parental autonomy support and growth mindset and between teacher autonomy support and growth mindset. Based on the theoretical and empirical perspectives reviewed above, we expected that parental autonomy support and teacher autonomy support would be each positively associated with growth mindset. Although autonomy support from different aspects might have distinct associations with growth mindset, we would not make a specific expectation due to the scarcity of research in this field.

Secondly, we aimed to explore whether sense of coherence mediated the association between parental autonomy support and growth mindset and between teacher autonomy support and growth mindset. According to the aforementioned theoretical perspectives (despite scarce empirical support), some possible explanations might be made. Sense of coherence might partially or fully mediate the association between parental autonomy support and growth mindset and between teacher autonomy support and growth mindset. To be specific, parental autonomy support and teacher autonomy support would be each positively associated with sense of coherence, which in turn would be positively linked to growth mindset.

Moreover, following prior research (e.g., Degol et al., 2018; Wang et al., 2020), there are some potential associations between sociodemographic variables and growth mindset. Given this, we regarded age, gender, and family socioeconomic status (SES) as possible covariates in this study.

## Materials and Methods

### Participants and Procedures

Before data collection, ethical approval was obtained from the Ethics Committee at Northwest Minzu University. Through personal networks, the authors contacted several public universities in different regions of China. Those regions were the southeast (i.e., Nanning, Guangxi Province), northwest (i.e., Lanzhou, Gansu Province), and north (i.e., Beijing) Mainland China. After negotiating with school principals and teachers, each of the potential participants was asked to fill out a set of online questionnaires (through a QR code generated by Wenjuanwang) in the presence of teachers during class hours. During the period of this survey, one participant was only allowed to submit their responses once, based on their electronic devices’ IP and login Wechat accounts (Jin et al., 2019). In the meantime, the duration of completing this survey was recorded automatically, and participants whose completion time was ±3 standard deviations of the mean completion time would be eliminated from the further course of data analysis. Moreover, we made some efforts to decrease the possibility of unintentionally skipping questionnaire items. This procedure is suggested by previous online-based research (e.g., Jin et al., 2019; Lan et al., 2019e). To be specific, skipping any questions would prompt a reminder, which allows participants to double check their responses before submission. However, participants were also informed that, if they felt uncomfortable or wanted to skip any specific research items, they were free to leave it blank or withdraw from the investigation. In addition, the participants’ rights (e.g., confidentiality and anonymity), standardized instructions, and informed content were highlighted at the beginning of this survey. Only on the condition that the participants approved these pieces of information would the follow-up survey start.

In this study, we failed to record the dropout rates of the participants. As indicated by prior research (O’Neil et al., 2003), online-based research usually exhibits high levels of dropout rates, posing specific challenges to this approach of data collection. Two aspects of the participants’ subjective experiences (interest in the questions and perceived burden while answering these questions) are regarded as essential factors that could potentially impact the dropout rates (Galesic, 2006). To reduce the dropout rates of the online-based survey, we took the following considerations into account when designing this online data collection. Firstly, we selected the short form of questionnaires to measure the study variables (see *Measure*) and kept the final research protocol as short as possible, which may minimize the participants’ burden. Secondly, all the participants were asked to complete this survey in the presence of their teachers during class hours. In China, the high participation rate is common due to high compliance with teacher/school authorities (Lan and Moscardino, 2019; Lan et al., 2019c,d). Thirdly, upon completion of this survey, the participants would be allocated a random cash reward ranging from 1 to 5 RMB based on the online survey system (Wenjuanwang). Prize draws have been demonstrated to be effective in decreasing the dropout rates (Bosnjak and Tuten, 2003), which have been successfully applied to the online-based survey of Chinese young adults (e.g., Lan et al., 2019a).

Finally, a total of 1,030 undergraduate students (62.4% females; *M*_age_ = 20.44, *SD* = 1.52) aged from 18 to 25 years were involved in the current study. Of these participants, 230 (22.3%) undergraduates were in their first year, 495 (48.1%) were in their second year, 251 (24.4%) were in their third year, and 54 (5.2%) were in their fourth year^2^^,^^3^. Moreover, 45.0% of students belong to the Han ethnic group (the ethnic majority in China) (Ma et al., 2019). In terms of parental education, the majority of fathers (66.9%) and mothers (77.5%) had completed primary or secondary school education. Most students (36.2%) reported that their family monthly income was relatively low (approximately US $400 per month)^4^.

Of these participants, 386 (37.4%) were recruited from public universities located in Beijing and Nanning and the remaining participants (*n* = 644, 62.6%) were recruited from public universities in Lanzhou. As a note, we did not report a specific percentage of participants from the universities located in Beijing and Nanning because those data collections occurred at the same time, and we failed to identify the locations of participants through the online system. Despite the lack of this valuable information (e.g., their hometowns and places of birth), the public universities in China are authorized to recruit students from all provinces based on a certain proportion^5^, according to the national college admission policy. This gives us a valuable opportunity to recruit the participants who originally come from different regions of China and to potentially increase the representativeness of the sample (Jin et al., 2019).

### Measures

All measures were selected based on their well-established psychometric properties and ease of comprehension (to intentionally reduce participants’ burden). We did not apply the standard translation–back-translation technique in this study based on the following considerations. Firstly, although back-translation is regarded as one of the best practices concerning the cross-cultural adaptation of the questionnaire, it is time-consuming and we have difficulties finding some bilingual experts who are proficient in both Chinese and English. Secondly, although prior research has predominantly used a back-translation procedure, much of the research relies on their close colleagues’ or friends’ assistance, which is prone to introducing errors and biases (Epstein et al., 2015a). Some researchers have criticized the back-translation approach due to the lack of rigorous evidence for this procedure in questionnaire adaptation (see a review by Epstein et al., 2015b). In the current study, we asked a group of psychology professors and students to discuss the questionnaires openly. During this discussion, we translated the questionnaires, resolved any ambiguities, and reached consensus for the face validity, acceptance, and cultural appropriateness of the questionnaires not available in Chinese. Moreover, we conducted confirmatory factor analyses (CFA) to assess the structural fit and calculated Cronbach’s alpha to evaluate the internal consistency of each questionnaire (de la Fuente et al., 2019). Based on the psychometric properties of the questionnaires (as reported below), it was indicated that this procedure might be appropriate for this study.

### Sociodemographic Characteristics

The participants were asked to indicate their age, ethnicity, grade level, parental education level, parental occupation, and family monthly income (adapted from Cui and Lan, 2020). The scores of parental education level, parental occupation, and family monthly income were standardized first and then summarized into a composite score, with the higher score indicating higher levels of family SES (Lan and Zhang, 2019; Lan and Radin, 2020).

### Perceived Parental Autonomy Support

We used a Chinese translation of the Perceived Parental Autonomy Support Scale (P-PASS) (Mageau et al., 2015) to measure the perception of parental autonomy support in undergraduate students. This scale contains 12 items and three dimensions: choice within certain limits (four items), rationale for demands and limits (four items), and acknowledgment of feelings (four items). One of the item examples is, “My parents gave me many opportunities to make my own decisions about what I was doing (choice within certain limits).” Participants were asked to rate these items by a seven-point scale ranging from 1 (*do not agree at all*) to 7 (*very strongly agree*). The average score of all items was calculated, with higher scores indicating higher levels of perception of parental autonomy support. Prior research has shown the good internal consistency of this scale in Chinese undergraduate students (Lan et al., 2019a). Results from the CFA exhibited good fit indices in the current sample [*χ*^2^/*df* = 7.94, *p* < 0.001, comparative fit index (CFI) = 0.96, Tucker–Lewis index (TLI) = 0.95, and root mean square error of approximation (RMSEA) = 0.08]. The Cronbach alphas of the P-PASS in this study were 0.85 for choice within certain limits, 0.86 for rationale for demands and limits, 0.90 for acknowledgment of feelings, and 0.93 for an overall score of parental autonomy support.

### Perceived Teacher Autonomy Support

Teacher autonomy support was measured by a Chinese translation of the 15-item Learning Climate Questionnaire (LCQ; Williams and Deci, 1996). The LCQ is a unidimensional questionnaire and consists of 15 items. One of the item examples is, “I feel that my head teacher provides me choices and options.” Participants rated each item based on a seven-point scale ranging from 1 (*strongly disagree*) to 7 (*strongly agree*). The average score of all items was calculated, with higher values representing higher levels of perception of autonomy support from head teachers. Prior research has shown acceptable psychometric properties of this scale in Chinese youth (e.g., Zhou et al., 2009; Lan and Zhang, 2019). In this study, the results from the CFA showed an acceptable model fit (*χ* ^2^/*df* = 12.29, *p* < 0.001, CFI = 0.90, TLI = 0.90, and RMSEA = 0.08); the Cronbach’s alpha was 0.93 in this study.

### Sense of Coherence

Sense of coherence was measured by the Chinese version of the Orientation to Life Questionnaire (short form; Antonovsky, 1987). This questionnaire comprised three dimensions – comprehensibility (five items), manageability (four items), and meaningfulness (four items) – which has been validated in Chinese undergraduate students (Bao and Liu, 2005). One of the item examples is, “Until now your life has very clear goals and purposes (meaningfulness).” Each item was rated on a seven-point scale ranging from 1 (*never*) to 7 (*always*). After reversing the scores of the five negatively worded items, an average score of all the items was calculated, with higher scores indicating a greater sense of coherence. Previous studies have shown good internal consistency of this questionnaire in Chinese undergraduate students (e.g., Bao et al., 2006). In the current study, the results from the CFA showed an acceptable model fit (*χ*^2^/*df* = 9.64, *p* < 0.001, CFI = 0.90, TLI = 0.88, and RMSEA = 0.08); the Cronbach’s alpha was 0.77. Although the structural fit of this questionnaire was not desirable enough, we retained the original factorial structure as this scale had been previously validated in Chinese college students (Bao and Liu, 2005).

### Growth Mindset

Growth mindset was measured by a Chinese translation of the Implicit Theory Scale (Dweck et al., 1995). This scale focuses on four domains: entity of intelligence (three items), entity of morality (three items), entity of the world (three items), and entity of personality (three items). One of the item examples is, “You have a certain amount of intelligence, and you cannot do much to change it (entity of intelligence).” Participants were asked to rate each item based on a six-point scale ranging from 1 (*strongly agree*) to 6 (*strongly disagree*). The mean score of all the items was calculated, with higher scores indicating a stronger belief that basic attributes are malleable. Prior research has shown good internal consistency in Chinese adults (using one subscale of the implicit theory scale; Chen et al., 2019). The results from the CFA showed an acceptable model fit (*χ*^2^/*df* = 4.68, *p* < 0.001, CFI = 0.98, TLI = 0.98, and RMSEA = 0.06); the Cronbach’s alpha was 0.96 in this study.

### Analytical Plan

Data analyses were performed using SPSS 21.0 (IBM Corp., 2012) and R software (R Core Team, 2018). In this study, missing values ranged from 0 to 0.7% in terms of the key study variables. Thus, full information maximum likelihood estimation (FIML) was applied to calculate the proposed model in the further course of analysis (Enders and Bandalos, 2001).

Before addressing our research questions, we conducted some exploratory analyses to assess the normality and the presence of outliers of the study variables (Lan and Wang, 2020). To be specific, the normality of the study variables was evaluated by skewness and kurtosis. Univariate skewness and kurtosis of 2.0 and higher were considered as criteria of non-normality (see George and Mallery, 2003; Lan and Wang, 2020). Moreover, to detect the presence of possible outliers in terms of the key study variables, we used the median absolute deviation (MAD; Leys et al., 2013). We did not use the traditional method (i.e., the mean ± 3 standard deviations) as this method has been criticized by many scholars (e.g., Leys et al., 2013). For instance, both the mean and standard deviation are particularly sensitive to possible outliers. In addition, analyses of the descriptive statistics (i.e., means and standard deviations) and zero-order correlations were performed to have a preliminary overview of the study variables.

To test our research hypotheses, we conducted structural equation modeling using package lavaan in R (Rosseel, 2012; R Core Team, 2018), following the procedures proposed by de la Fuente et al. (2020). The goodness-of-fit was tested with chi-square (*χ*^2^; a non-significant value corresponds to an acceptable fit), but *χ*^2^ are sensitive to the sample size and degree of freedom (Schermelleh-Engel et al., 2003). In view of this, the *χ*^2^ was complemented by examining other indices (i.e., CFI, TLI, and RMSEA) that depend on a conventional cutoff. The following cutoff values indicate an acceptable model fit: CFI/TLI > 0.90 and RMSEA < .08 (Schreiber et al., 2006). In addition, indirect effects were tested using the bootstrapping approach considering a 95% confidence interval (bootstrap replications: 5,000; Preacher and Hayes 2008). If the confidence interval did not contain zero, these effects were considered statistically significant (Jin et al., 2019; Cui and Lan, 2020).

## Results

### Preliminary Analysis

Descriptive statistics for the study variables are reported in Table 1. Based on the values of skewness and kurtosis, all the study variables were demonstrated to be normally distributed. Following the procedure proposed by Leys et al. (2013), we first calculated the median of the key study variables (5.16 for parental autonomy support, 4.85 for teacher autonomy support, 4.15 for sense of coherence, and 4.66 for growth mindset). The criteria of the median ±2.5 times were used to detect possible outliers of the study variables. The results exhibited that no extreme outliers were identified in this study (the MAD scores of the study variables ranged from −6.17 to 2.73 for parental autonomy support, from −5.71 to 3.19 for teacher autonomy support, from −3.76 to 3.88 for sense of coherence, and from −4.51 to 3.13 for growth mindset).

**Table 1:** Descriptive statistics and bivariate correlations of the study variables for Chinese undergraduate students.

S.No.	Variables	*M*	SD	Range	Skewness	Kurtosis	1	2	3	4	5	6	7
1.	PAS	5.11	1.12	1–7	−0.59	0.33	–						
2.	TAS	4.82	0.97	1–7	−0.30	0.22	0.51^***^	–					
3.	SOC	4.18	0.72	1–7	0.19	0.23	0.23^***^	0.32^***^	–				
4.	GM	4.35	1.28	1–6	−0.19	−0.91	−0.01	0.02	0.17^***^	–			
5.	Age	20.44	1.52	18–25	–	–	0.02	−0.01	−0.02	−0.27^***^	–		
6.	Gender^a^	–	–	1–2	–	–	0.03	0.04	−0.13^***^	−0.21^***^	−0.03	–	
7.	SES	0.00	3.24	−5.93 to 13.13	–	–	−0.04	−0.01	−0.02	−0.30^***^	−0.14^***^	0.03	–

*N* = 1,030. PAS, parental autonomy support; TAS, teacher autonomy support; SOC, sense of coherence; GM, growth mindset; SES, socioeconomic status.

*** *p* < 0.001.

a Coded as 1 = male, 2 = female.

In terms of bivariate correlations among the study variables, parental autonomy support was positively related to sense of coherence (*r* = 0.23, *p* < 0.001), teacher autonomy support was positively associated with sense of coherence (*r* = 0.32, *p* < 0.001), and sense of coherence was positively related to growth mindset (*r* = 0.17, *p* < 0.001). In terms of covariates, age was negatively associated with growth mindset (*r* = −0.27, *p* < 0.001), females reported higher levels of sense of coherence (*r* = −0.13, *p* < 0.001) and growth mindset (*r* = −0.21, *p* < 0.001) than did males, and undergraduate students with higher family SES reported lower levels of growth mindset (*r* = −0.30, *p* < 0.001) as compared with those from lower family SES.

### Structural Equation Model Analysis

We first estimated the model fit of the measurement model that included four latent variables of parental autonomy support, teacher autonomy support, sense of coherence, and growth mindset. The parental autonomy support latent variable has three subscales, the sense of coherence latent variable has three subscales, and the growth mindset latent variable has four subscales. For teacher autonomy support, three random parcels were established (Sass and Smith, 2006). The measurement model revealed an acceptable model fit to the data: *χ* ^2^/*df* = 5.10, *p* < 0.001, CFI = 0.97, TLI = 0.96, and RMSEA = 0.06.

Secondly, we built the direct effects model, which addressed whether both parental autonomy support and teacher autonomy support had direct effects on growth mindset. The direct effects model demonstrated an acceptable model fit to the data: *χ* ^2^/*df* = 5.46, *p* < 0.001, CFI = 0.96, TLI = 0.96, and RMSEA = 0.07. However, the direct effects model showed that neither parental autonomy support (*B* = −0.04, SE = 0.04, *t* = −0.86, *p* = 0.38) nor teacher autonomy support (*B* = 0.02, SE = 0.05, *t* = 0.50, *p* = 0.61) was a significant predictor of growth mindset.

**Figure 1:**
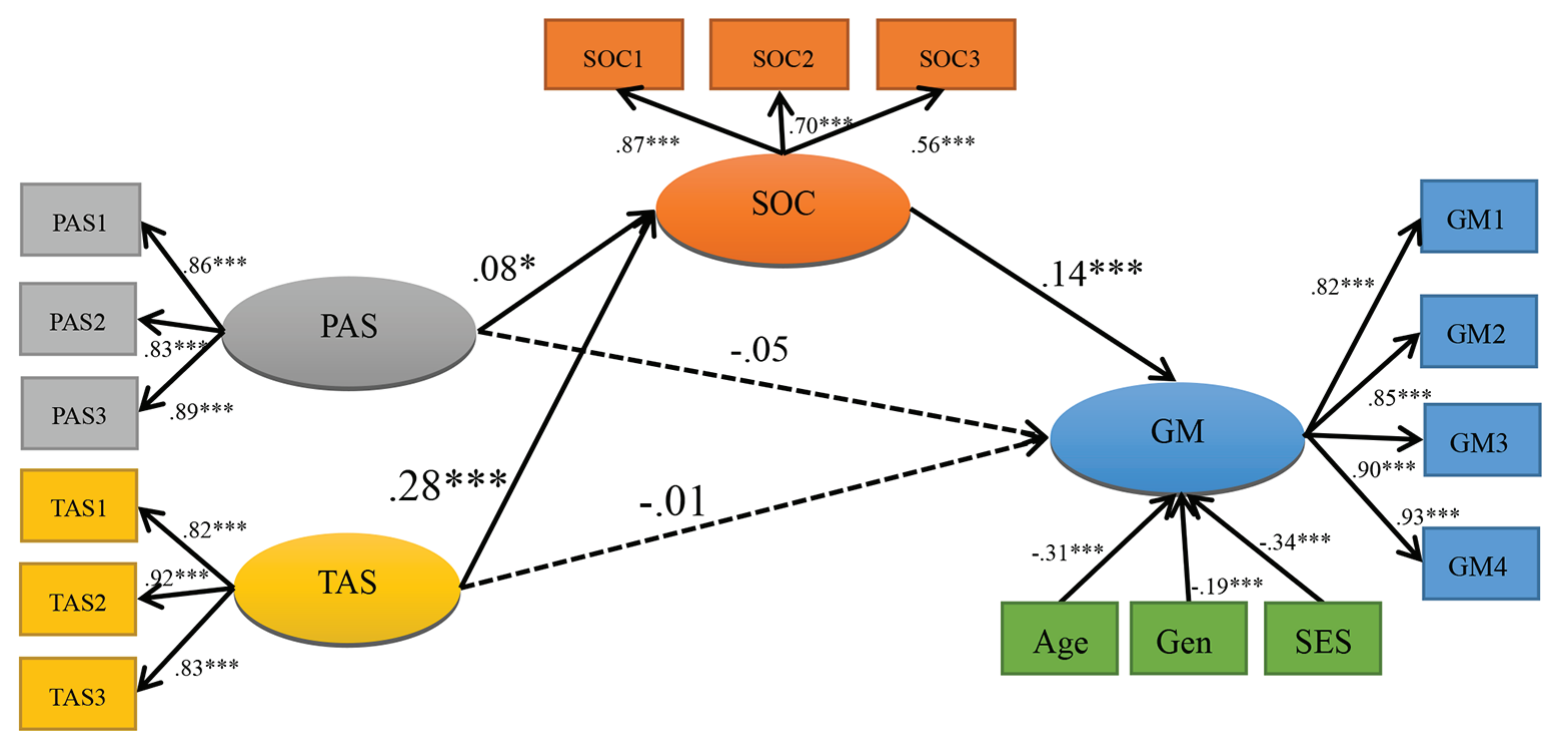
Structural model for growth mindset. *N* = 1,030. PAS, parental autonomy support; TAS, teacher autonomy support; SOC, sense of coherence; GM, growth mindset; Gen, gender; SES, socioeconomic status. The coefficients shown are standardized path coefficients. Statistically significant and insignificant paths were presented by *solid lines* and *dotted lines*, respectively. This model explained 24.8% of the variance in growth mindset. ^*^ *p* < 0.05 and ^***^ *p* < 0.001.

Thirdly, after controlling for age, gender, and family SES, we inserted sense of coherence as a mediating variable between parental autonomy support and growth mindset and between teacher autonomy support and growth mindset. The multiple indirect effects model depicted in Figure 1 demonstrated a good fit: *χ* ^2^/*df* = 4.27, *p* < 0.001, CFI = 0.97, TLI = 0.96, and RMSEA = 0.06.

Lastly, we adopted the bias-corrected bootstrap method to evaluate the significance of the indirect effects model. The results showed that the total indirect effect of parental autonomy support on growth mindset through sense of coherence was 0.04 (95% CI = 0.02–0.05) and that of teacher autonomy support was 0.06 (95% CI = 0.03–0.09). Moreover, the direct effect of parental autonomy support on growth mindset was −0.04 (95% CI = −0.11 to 0.01) and that of teacher autonomy support was −0.02 (95% CI = −0.09 to 0.05). Confidence intervals for the direct effects included zero, suggesting that the direct effects were not significant.

### Alternative Model Analysis

Given that sense of coherence is assumed to be a stable trait (Schnyder et al., 2000), we also tested whether it might moderate the association between perceived autonomy support and growth mindset. However, the results did not support such an expectation: sense of coherence did not moderate the associations between parental autonomy support and growth mindset (*B* = −0.01, SE = 0.01, *t* = −1.40, *p* = 0.16) and between teacher autonomy support and growth mindset (*B* = −0.01, SE = 0.01, *t* = −1.22, *p* = 0.22).

Moreover, although we tested the conceptual model based on some theoretical and empirical considerations, we had to acknowledge that this proposed model was examined in an exploratory fashion as these variables had not been studied previously. For instance, we cannot determine the causal association between perceived autonomy support and growth mindset. It is conceivable that undergraduate students who hold a strong growth mindset may tend to perceive that they receive more autonomy support from their parents and teachers. In this perspective, we performed another alternative model to potentially rule out this possibility (at least in the current sample). To be specific, we reran the proposed structural equation model by reversing the independent and dependent variables (i.e., regarding growth mindset as an independent variable and parental autonomy support and teacher autonomy support as dependent variables). The results showed a relatively worse model fit, *χ* ^2^/*df* = 6.99, *p* < 0.001, CFI = 0.94, TLI = 0.92, and RMSEA = 0.08 (*R* ^2^ = 7.4% for parental autonomy support and 13% for teacher autonomy support), as compared with the proposed model.

## Discussion

Although prior research has predominantly investigated the correlates of growth mindset, little is known about its contextual and individual antecedents among undergraduate students. To fill this research gap, the current study examined the association between perceived autonomy support and growth mindset and explored whether sense of coherence mediated this association. Our findings showed that sense of coherence fully mediated the association between parental autonomy support and growth mindset and between teacher autonomy support and growth mindset. To be specific, parental autonomy support and teacher autonomy support were each positively associated with sense of coherence, which in turn was positively related to growth mindset among Chinese undergraduate students.

Our research purpose was to explore the association between perceived autonomy support and growth mindset. Distinct from our expectation, the results did not show a significant direct association between perceived autonomy support and growth mindset. This finding is in line with previous studies (e.g., Shen et al., 2009; Jungert and Koestner, 2015), highlighting the mediating or moderating processes involved in the association between autonomy support and the adaptive skills of individuals. Instead of a significantly direct linkage, autonomy support has been indirectly or interactively linked with other individual characteristics (e.g., basic psychological needs satisfaction and cognitive orientation; Jungert and Koestner, 2015; Yu et al., 2016), which in turn impacts individuals’ adaptive skills.

In this study, the results revealed that sense of coherence fully mediated the association between parental autonomy support and growth mindset and between teacher autonomy support and growth mindset. These findings suggest that sense of coherence is an essential mediator between autonomy support and growth mindset. One possible interpretation is that autonomy support can foster optimal psychological function and imbue life with a sense of purpose (Ryan and Deci, 2000; Vansteenkiste et al., 2008). Moreover, having a strong sense of coherence allows students to integrate critical elements of their life as a whole (comprehensibility and manageability) and their efforts to maintain internal harmony and ultimate purpose (meaningfulness; Krok, 2015).

Furthermore, by simultaneously investigating autonomy support from both parents and teachers, the current findings showed that teachers exhibited a stronger effect on sense of coherence than did parents. This may be because undergraduate students spend most of their time in college and have more interactions with teachers. In this context, undergraduate students are characterized by decreased parental reliance, and teachers are more available than parents to provide direct and significant autonomy support. Likewise, such a finding can be interpreted by Chinese cultural values. For example, the educational system in China has been regarded as highly hierarchical, and teachers at school are treated as essential authorities (Helwig et al., 2003; Yu et al., 2016). In a sense, students usually show utmost respect and obedience to the authority of teachers at school. Given the salient role of teachers in undergraduate students’ academic life or beyond, teachers may become the more prominent autonomy-supporting figures on their optimal functions and adaptive skills.

According to these significant findings, the current study may contribute to the literature in several manners. Firstly, by using a relatively larger sample size (recruiting from multiple sites in China), we address the contextual and individual antecedents of growth mindset, which provides some solid evidence for intervention or prevention programs facilitating growth mindset among undergraduate students. Secondly, we further enrich the SDT theory in a collective society. Although traditional Chinese parents and teachers are assumed to be less autonomy-supportive and more controlling, contemporary Chinese parenting and teaching ideologies have been possibly adapted in response to dramatic sociocultural changes (Liew et al., 2014; Lan and Zhang, 2019). Perhaps, many parents and teachers still retain their traditional styles, but it seems undeniable that they also progressively realize the vital role of being autonomy-supportive on individuals’ development when interacting with their children/students. Moreover, we simultaneously investigate the role of parental autonomy support and teacher autonomy support on growth mindset in one single study. The findings suggest that teachers may have a stronger effect than parents on undergraduate students’ sense of coherence, which in turn fosters their growth mindset. Thirdly, we explore the possible underlying mechanism of the association between perceived autonomy support and growth mindset, which may further catalyze the theory-based evidence development in the growth mindset literature.

### Limitations and Future Research Directions

Along with these significant findings, several limitations need to be acknowledged in the present research. Firstly, this study is highly constrained by using a cross-sectional design to document a mediation model. This is because such a design cannot infer directionality among the study variables and potentially generates biased estimates of the parameters (Maxwell and Cole, 2007). Given the exploratory nature of this study, future studies should examine the associations longitudinally to look for reciprocal or reverse effects of these variables.

Secondly, the current study focuses on hierarchical relationships only (i.e., between parents and their children and between teachers and students), but fails to incorporate autonomy support from close interpersonal relationships (e.g., Deci et al., 2006; Jungert et al., 2013; Ratelle et al., 2013). Moreover, unpacking the differential roles of paternal and maternal support could also be interesting, given the potential father–child and mother–child interaction differences with each Chinese family (Cui and Lan, 2020; Lan and Wang, 2020). Given these, further investigation may consider examining more possible autonomy-supportive figures in undergraduate students’ daily life so as to have a fine-grained understanding of the association between perceived autonomy support and growth mindset.

Thirdly, the current study relies solely on self-report measurements, which may inflate the study associations. For example, as documented by Podsakoff et al. (2003), research using self-report questionnaires may be biased by common method variance and social desirability. Although the use of self-report tools enables us to assess students’ perception of autonomy support, robust support for the current model could be further confirmed by using a multimethod approach (e.g., structural interviews or observations) and/or a multi-informant approach (parent‐ and teacher-rated) to evaluate these study associations.

Fourthly, although the questionnaires in this study (i.e., parental autonomy support, teacher autonomy support, and growth mindset) have shown proper psychometric properties, these questionnaires have not been validated in Chinese undergraduate students. For example, since we use the translated versions of these measurements (but the back-translation procedure is not administrated in this study), it may cause measurement variance problems between the original version of the questionnaire and the translated version (Widaman and Reise, 1997; Davidov et al., 2014). In order to make an appropriate adaption of the questionnaire in the Chinese culture, we highly recommend that future studies may validate these questionnaires, providing solid evidence in terms of cultural adaptation and measurement invariance.

Finally, despite several advantages (e.g., simple and easy to operate, affordable, and fast access to the possible participants) and the increasing popularity of using the online-based survey in psychology research, it also involves some potential drawbacks (e.g., under-coverage and self-selection bias; Bethlehem, 2010). For example, since only those who complete the questionnaires are recorded, some scholars argue that those participants may not be representative of the study population (Schonlau et al., 2009). In terms of this under-coverage issue, we are less concerned about it in the current sample. Based on a recent report released by China Internet Network Information Center (2020), there are approximately 904 million Internet users, and the prevalence of Internet use is expected to be prevalent in Chinese undergraduate students (Yao et al., 2014). Moreover, we administrate this survey during class hours in the presence of responsible teachers, which may potentially reduce the self-selection bias. Despite these efforts, we cannot entirely exclude these biases. Thus, future studies may consider conducting online-based survey based on a mixed-mode strategy (Schonlau et al., 2003). For instance, researchers can intentionally recruit one part of the final sample that is unwilling or unable to be involved in an online-based survey. Likewise, the use of propensity scores to balance those biases is highly recommended for further course of research based on an online survey mode (see Schonlau et al., 2009).

## Conclusions

Although traditional Chinese culture requires parents and teachers to discipline their children and students restrictively, in this study, we stress the importance of being autonomy-supportive in the family and school contexts, which may promote a strong sense of growth mindset. In particular, considering that undergraduate students spend most of the time in college, teacher autonomy support should be highly emphasized in teaching and instructional design. Furthermore, the current study provides some preliminary evidence about the underlying mechanism of the linkage between perceived autonomy support and growth mindset: sense of coherence fully mediates (does not moderate) this association. This indicates that autonomy-supportive parents and teachers may enable undergraduate students to develop a comprehensible, manageable, and meaningful orientation, which in turn enables them to believe the malleability of individuals’ attributes and exert more effort to achieve it.

## Data Availability Statement

The datasets generated for this study are available on request to the corresponding author.

## Ethics Statement

The studies involving human participants were reviewed and approved by Northwest Minzu University. Written informed consent to participate in this study was provided by the participants.

## Author Contributions

CM conceived and drafted the manuscript. YM was the principal investigator of this study. XL performed the statistical analyses and critically revised the manuscript. All authors contributed to the article and approved the submitted version.

### Conflict of Interest

The authors declare that the research was conducted in the absence of any commercial or financial relationships that could be construed as a potential conflict of interest.

## Footnotes

^1^In the present study, we focused on head teachers who have broad responsibilities for undergraduate students’ social, behavioral, academic, and daily life activities in college (Chen et al., 2003). Therefore, students usually have more contact with head teachers, who are more available to provide proper support.

^2^Generally speaking, it takes a 4-year period for Chinese undergraduate students in public universities (except for medical students) to be awarded academic degrees.

^3^Although it is beyond our research question, we also performed a multiple-group structural equation modeling using package lavaan in R (Rosseel, 2012; R Core Team, 2018) to assess whether the study associations were invariant across four grade levels. To do this, we followed the procedures proposed by well-established empirical studies (e.g., Behnke et al., 2011; Guo, 2020). Firstly, a four-group structural equation modeling was evaluated, in which all study associations were freely estimated; the results demonstrated good fit indices (*Ï‡*^2^ = 406.32, *df* = 95, *p* < 0.001, CFI = 0.966, TLI = 0.958, RMSEA = 0.057). Secondly, a constrained four-group structural equation modeling was conducted, in which all study associations were set to be equal across four grades; the results showed good fit indices (*Ï‡*^2^ = 802.65, *df* = 434, *p* < 0.001, CFI = 0.957, TLI = 0.954, RMSEA = 0.059). Thirdly, we compared the model fit indices of the unconstrained model with the constrained model; the differences between CFI and RMSEA were both lower than 0.01 (Cheung and Rensvold, 2002), which indicated that the study model was invariant by the grade levels. It is noteworthy that we did not rely on the results of the *Ï‡*^2^ difference test as this approach has been demonstrated to be quite sensitive to sample size (Cheung and Rensvold, 2002), and the number of students from the fourth grade was only 54 in the current study.

^4^Based on a recent report released by the National Bureau of Statistics of China (2019), the average monthly income was approximately US $500 urban households.

^5^The high admission proportion is usually given to those students who come from the same province. For example, a university in Lanzhou is entitled to recruit a high percentage of students who come from Gansu Province. This is partially due to the cultural values highlighting family relations and personal networks in China (Bond, 2010). In this context, students do not usually wish to go to other provinces to pursue higher education because of the geographical distance between them and their family members. Given the considerable regional differences in China (Talhelm et al., 2014; Lan and Wang, 2019), we contacted public universities located in different regions of China so as to recruit a variety of samples.
